# Advanced hidradenitis suppurativa (HS) as a sentinel manifestation of undiagnosed diabetes in a previously healthy male: a case report

**DOI:** 10.1097/MS9.0000000000003850

**Published:** 2025-09-11

**Authors:** Amna Amjad, Eeman Amjad, Marium Saeed, Yashal Naveed, Maariya Shereen, Saira Naseem, Asad Ali Ahmed Cheema

**Affiliations:** a Department of Medicine, FMH College of Medicine and Dentistry, Lahore, Pakistan; b International School of Medicine, International University of Kyrgyzstan, Bishkek, Kyrgyzstan

**Keywords:** case report, diabetes mellitus, hidradenitis suppurativa, Hurley stage III, obesity, pilosebaceous–apocrine unit, V–Y flap reconstruction

## Abstract

**Introduction and importance:**

Hidradenitis suppurativa (HS) is a chronic, long-term inflammatory disorder of the skin that affects pilosebaceous–apocrine units. Follicular occlusion within these units leads to follicular rupture, which triggers immune response, followed by bacterial colonization, and ultimately chronic sinus tract formation presenting with painful lumps, draining sinuses, and scarring. It is often diagnosed late and is associated with metabolic comorbidities, such as obesity and diabetes. This case of HS was the sentinel manifestation of previously undiagnosed type 2 diabetes in a seemingly healthy 47-year-old male with bilateral axillary Hurley stage III disease.

**Presentation of case:**

A 47-year-old obese male presented with 1.5 years of bilateral axillary nodules, draining sinuses, scarring, and mild constitutional symptoms, more marked on the right side. Labs revealed leukocytosis and an elevated HbA1c of 12.8%, confirming the status of diabetes. Treatment involved right-sided wide excision with V–Y flap reconstruction and left-sided incision and drainage under IV cefazolin and metronidazole. Post-operative glycemic control was achieved by insulin therapy and analgesia for symptom relief.

**Clinical discussion:**

This case highlights HS as a systemic inflammatory condition. Although the patient had no smoking history or family history of HS, obesity may have contributed to severity of disease. Prior literature shows that HS patients have two to three times higher odds of type 2 diabetes mellitus (T2DM). The Hurley stage III diagnosis underscores the importance of essential metabolic screening, especially in older or obese individuals.

**Conclusion:**

HS is a marker of metabolic disease, whether presented in early or late stages. A multidisciplinary approach can optimize early diagnosis and recovery. Routine screening of T2DM in severe HS cases is necessary to prevent complications, recurrence, and to improve patient outcomes.

## Introduction

Hidradenitis suppurativa (HS) is a debilitating chronic inflammatory condition of the follicular epithelium characterized by the presence of nodules and abscesses in apocrine units. These units, when occluded, undergo rupture that triggers an immune response, followed by bacterial colonization and eventual chronic sinus tract formation in the intertriginous areas of the body[1]. It is associated with multiple comorbidities like metabolic syndrome, Inflammatory Bowel Disease (IBD), and obesity[2]. Prevalence of the disease ranges from 0.00033% to 4.1%, making it not an uncommon occurrence[3]. HS occurs in areas of skin that contain apocrine glands, including axillae and groins, but also the scalp, breast, chest, and perineum[4].

**HIGHLIGHTS**

- Hidradenitis suppurativa (HS) may serve as a clinical marker for undiagnosed metabolic disease, particularly type 2 diabetes mellitus.
- This case demonstrates HS as a sentinel presentation of diabetes in a patient without prior metabolic or family history.
- Early recognition and metabolic screening in advanced HS cases can improve outcomes and prevent complications.

The first description of the disease dates back to 1839, but the connection with the sweat glands was made by Aristide Verneuil in 1854[5]. HS is a part of a follicular occlusion tetrad, including acne conglobata, cellulitis of the scalp, and pilonidal sinus[6]. The condition is associated with pain, drainage, scarring, malodor, and negative psychological implications[7].

To guide clinical assessment and management, HS is classified using the Hurley staging system. *Stage I* involves single or multiple abscesses without sinus tract formation or scarring. *Stage II* is characterized by recurrent abscesses with sinus tract formation and scarring. *Stage III* indicates diffuse or extensive involvement, with multiple interconnected sinus tracts and abscesses spanning the entire affected region[8].

Lifestyle factors, including tobacco smoking and obesity, are the major contributors to the development of HS[9]. The diagnosis, however, is clinical, requiring no biological or pathological tests for confirmation. Treatment options involve medications such as antibiotics, steroids, hormonal therapies, and pain management. If left untreated, surgical procedures are performed, including tunnel uncovering, laser therapy, surgical removal of affected skin, and incision and drainage[10]. This case report has been reported in line with the SCARE checklist 2025[11].

A brief literature search was conducted in PubMed and Google Scholar using the terms “hidradenitis suppurativa,” “diabetes,” “obesity,” and “surgery.” Priority was given to peer-reviewed English-language articles, systematic reviews, and clinical guidelines.

## Presentation of case

This report highlights the case of a 47-year-old married Asian male who presented to the surgical outpatient department with a history of multiple wounds in the axillary region persisting over the past 1.5 years and was admitted to the surgical ward for further evaluation and treatment. The patient initially noticed a lump in his axilla. It gradually progressed into multiple chronic recurrent nodular lesions that were asymmetrical and affecting both axillae. The condition was exacerbated by sweating and heat but was not associated with psychological stress. The lesions frequently ruptured, discharging odorless pus with mild bleeding, followed by scarring and fibrosis. With inadequate treatment, the frequency of flare-ups increased and led to the formation of sinus tracts and tunnels between lesions. The episodes of purulent discharge were often accompanied by low-grade fever, which resolved once the pus drainage ceased.

The patient used Panadol for fever relief with partial improvement. He had previously sought treatment at a local clinic where he was prescribed antibiotics and analgesics, which only provided temporary relief. He began wearing loose clothing to minimize friction and irritation of the affected areas. Pain was associated with flare-ups and purulent discharge, significantly interfering with daily activities such as upper limb movements, motorcycle riding, and work performance. The pain was aggravated by physical activity and mildly relieved by analgesics. There was insignificant weight loss or any dietary changes with normal appetite, no history of chronic cough and orthopnea, joint pain, swelling, and no features suggestive of autoimmune disease. No known chronic medical condition. No history of blood transfusion; however, the patient had donated blood once. No known drug allergies were reported, and no regular medications were being taken. No family history of similar condition or autoimmune diseases. No smoking or alcohol use.

**Figure 1.**
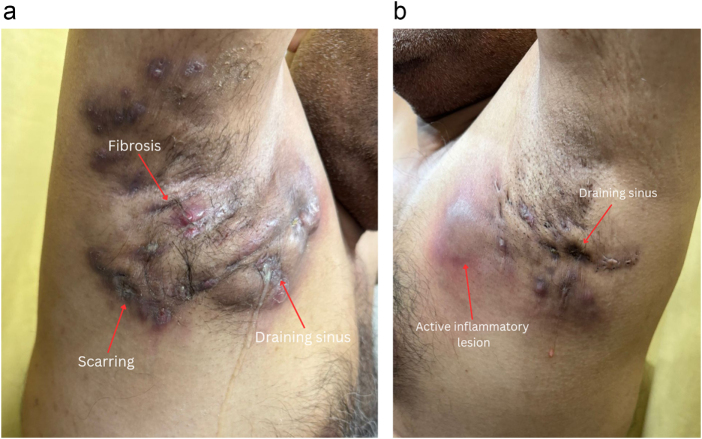
(a) Right axilla showing multiple nodular lesions with draining sinuses, scarring, and fibrosis. (b) Left axilla with less extensive but active inflammatory lesions and sinus tracts.

On examination, the patient was afebrile and lying comfortably in bed. Vital signs were within normal limits: Blood pressure 130/90 mmHg, pulse 82 beats per minute, body mass index (BMI) was 33 kg/m^2^. The Glasgow coma scale score was 15/15, indicating full consciousness. Chest examination revealed bilateral clear breath sounds. Local examination showed multiple draining sinuses in both axillae, discharging foul-smelling purulent material (Fig. 1a,b).

The diagnosis was made on a clinical basis supported by characteristic presentation with no evidence of systemic infection; no biopsy or imaging was considered necessary due to the typical nature of the lesion and chronic history. The condition identified was hidradenitis suppurativa, likely Hurley stage III based on sinus formation and bilateral involvement.

Laboratory investigations revealed leukocytosis with a total leukocyte count of 12.28 × 10^3^/µL and neutrophilia (9.09 × 10^3^/µL), suggesting ongoing infection. Mild lymphopenia was noted (19.5%). Hematological indices showed slightly low Mean Corpuscular Volume (MCV) and Mean Corpuscular Hemoglobin (MCH), although hemoglobin and red blood cell counts were within normal limits. Platelets were mildly elevated. Renal function tests, liver function tests, serum electrolytes, and urinalysis were in normal range, except for globulin, which was reported in high concentration. Screening for viral markers (Hepatitis B Surface Antigen (HBsAg), anti-HCV, and anti-HIV) was non-reactive. Notably, HbA1c was markedly elevated at 12.8%, indicating poorly controlled diabetes mellitus. The coagulation profile was in the normal range.

**Figure 2.**
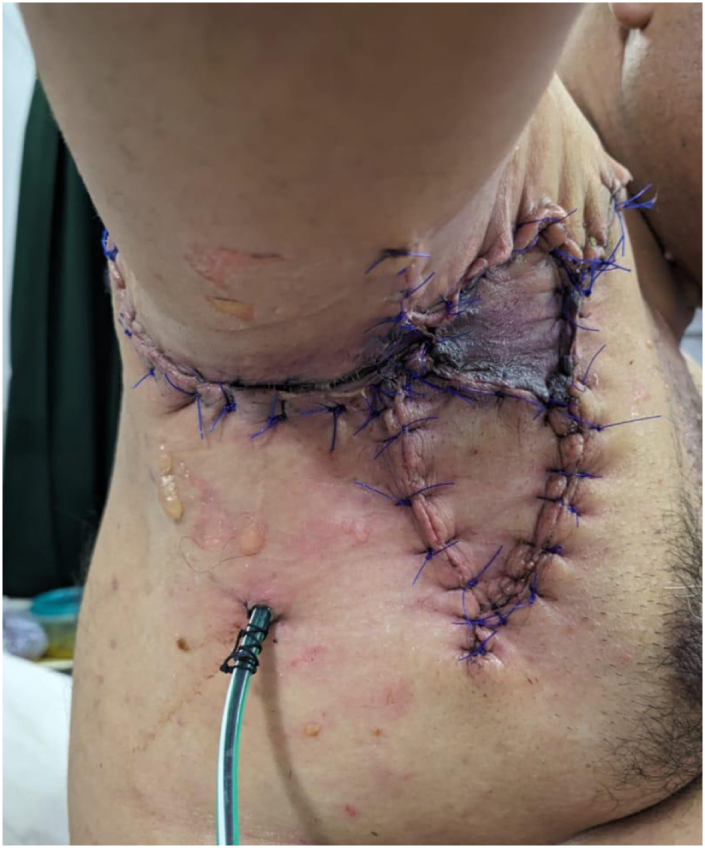
Right axilla post closure showing surgical drain and sutured V–Y flap.

After ensuring adequate pre-operative control and stabilization, bilateral axillary sinus excision and drainage were performed. On the right side, a 5 × 7 scarred tissue with multiple sinuses was excised using an elliptical incision. The sinus tracts were removed, and infected tissue was debrided. V–Y advancement flaps were mobilized for wound closure, and a surgical drain was placed to monitor for infection or bleeding (Fig. 2).

On the left side, a 3 × 3 axillary abscess was noted. Incision and drainage were conducted along with probing of sinuses, curettage, and complete drainage. The wound was left open for evaluation and healing (Supplemental Digital Content Fig. 1, available at: http://links.lww.com/MS9/A937).

Excised tissue from the right axilla was sent for histopathological analysis (Supplemental Digital Content Fig. 2, available at: http://links.lww.com/MS9/A938). The histopathology report grossly described it as a specimen of right axilla consisting of irregular fibrofatty tissue fragments. The largest fragment measures 8.7 × 6.5 × 2.5 cm, with an attached skin ellipse measuring 6.3 × 3.0 cm. The smaller fragment measures 5.9 × 3.5 × 3.0 cm, with an attached skin ellipse measuring 5.0 × 3.0 cm. Serial sectioning reveals grey and white areas. The microscopy findings showed sections of skin with underlying multiple sinus tracts lined by inflamed granulation tissue and dense mixed predominantly chronic inflammation. Microabscess formation and fibrosis surrounding the tract are also seen. One of the tracts shows epithelial lining. Dilated benign sweat glands adjacent to the tract are noted. No granuloma or malignancy was found, making the diagnosis consistent with HS. No intraoperative cultures were obtained, which limits microbiological insight into this patient’s disease.

Post-operatively, the patient was managed with IV antibiotics, including cefazolin for gram-positive coverage and metronidazole for anaerobic organisms commonly involved in HS. Analgesia was provided by intramuscular diclofenac to control pain and reduce inflammation. Proton pump inhibitors were used to prevent Non-Steroidal Anti-Inflammatory Drug (NSAID)-induced gastritis. Metoclopramide and antiemetics were administered to control postoperative nausea and vomiting. Due to significantly elevated HbA1c, insulin therapy was initiated. Subsequent blood sugar readings indicated reasonable glycemic control (BSR was 128 g/dL). The patient was advised to restrict arm movement to reduce tension on surgical flaps and minimize mechanical disruption to healing sites. Daily Complete Blood Count (CBC) monitoring was performed for early detection of post-operative complications. Follow-up of this patient focused on glycemic control with insulin initiation, but longer-term endocrinology monitoring would have provided stronger insight into the trajectory of diabetes management after surgery. The patient was reviewed at 1, 2, and 3 months postoperatively, with satisfactory wound healing, no recurrence, and continued glycemic control.

Patient’s perspective: Before coming to the hospital, I tried using other remedies with the hope that this will resolve on its own. However, with time the recurrent abscesses with episodes of fever made me carry tablets with me. After surgery, I feel much better and can do my daily activities without constant irritation.

## Discussion

This case report illustrates the complex bidirectional relationship between cutaneous inflammation and systemic disease, presenting an advanced case of HS in a patient later diagnosed with type 2 diabetes mellitus (T2DM) which is a proven comorbidity and was eventually discovered in this patient after the disease had already progressed to Hurley stage III in one axilla. HS is now increasingly being recognized as chronic systemic inflammatory condition associated with metabolic and cardiovascular associations, including obesity, diabetes, dyslipidemia, and metabolic syndrome[12].

Recent studies show a significant link between HS and metabolic comorbidities, especially T2DM. Several large cohort studies and meta-analyses have reported that patients with HS have two to three times higher odds of T2DM compared with the general population, with pooled odds ratios ranging from 2.1 to 2.8 (95% CI, 1.7–3.4) compared to non-HS controls^[13,14]^. In an extensive cross-sectional study conducted in the United States, T2DM was found to be more common in patients with HS (24.8%) than in those without HS (15.6%) and after controlling for age, sex, race, obesity, smoking, and comorbidities, the study also revealed that HS patients had 1.75 times higher unadjusted odds of having T2DM than controls[15]. Results from another meta-analysis reinforced the correlation of HS with DM, while also highlighting obesity and smoking as potential risk factors. Specifically, the odds of T2DM in HS patients were almost three times higher [OR 2.78 (95% CI 2.23–3.47)], while obesity and smoking were also significantly associated with increased risk [ORs 2.48 (95% CI 1.64–3.74), and 3.10 (95% CI 2.60–3.69), respectively][16]. These findings suggest that HS itself may contribute to metabolic dysfunction through the action of pro-inflammatory cytokines such as TNF-α, IL-1β, IL-17, and interferon-γ which are implicated in both insulin resistance and HS pathogenesis[9].

Despite increased clinical awareness, HS is still difficult to diagnose on time. In our case, the patient’s reliance on local treatment and over-the-counter painkillers contributed to a significant diagnostic delay, leading to a more severe disease presentation. The diagnosis of HS is often delayed by 3–12 years, and patients, particularly those who are non-white, female, or have more severe symptoms, may experience 3–4 misdiagnoses before receiving an accurate diagnosis[17]. Diagnostic delays in HS are often due to unfamiliarity of physicians with the condition and frequent misdiagnosis, despite the presence of an established diagnostic criterion. Younger patients and non-smokers face longer delays, and many consults multiple doctors before receiving an accurate diagnosis[18]. Early symptom onset, atypical lesion sites (like breasts and thighs), follicular phenotype, and obesity correlate with later diagnosis, possibly due to delayed care-seeking or misattribution of symptoms[19] and delayed diagnosis results in many serious consequences related to disease severity, surgical burden, comorbidities, psychological health, and work life. According to a clinical trial, longer delays were significantly associated with higher Hurley stage at diagnosis and a greater number of surgically treated sites, particularly abscess incisions. Patients with the most extended delays also had more comorbid conditions, especially musculoskeletal disorders and mental health issues, with a clear link to higher depression scores on the Hospital Anxiety and Depression Scale (HADS) scale. Additionally, diagnostic delay had a negative impact on patients’ professional lives. Those with longer delays reported more days of work disability, highlighting the socioeconomic burden of delayed HS recognition and diagnosis[18].

Although T2DM screening is already recommended for obese adults by USPSTF guidelines[20], the presence of HS should alert clinicians to an even higher risk profile. Severe HS (Hurley stage III) has been shown to carry a more than fivefold increased risk of diabetes compared with stages I and II[21].

The strong association between the two conditions becomes significant in severe cases. It has been demonstrated that patients with Hurley stage III HS are 5.3 times more likely to develop diabetes than patients with Hurley stages I and II. The metabolic-inflammatory nature of HS is further reinforced in those with elevated BMI, aligning with our patient, which increases this risk and underscores the importance of systematic metabolic screening in HS, particularly in older adults and those with elevated BMI should undergo routine diabetes screening using the HbA1c or fasting blood glucose test in light of these findings[22]. To facilitate early detection and maximize long-term results, such screening procedures must be incorporated in the clinical guidelines for the management of HS. A recent clinical study demonstrated that HS patients have significantly increased insulin resistance (Homeostatic Model Assessment of Insulin Resistance, HOMA-IR) and disrupted Spondyloarthritis Research Consortium of Canada Enthesitis Index – Genital Region (SPINAG-R) scores compared to controls[23]. These metabolic disturbances were related to inflammatory markers like haptoglobin, further strengthening the hypothesis that chronic inflammation in HS contributes to metabolic dysfunction.

HS should be considered as a dermatological marker for potential metabolic disease. Early identification of comorbidities allows better disease control combined with effective treatment. HS has a significant disease burden beyond the skin, including metabolic, cardiovascular, endocrine, gastrointestinal, rheumatologic, and psychiatric disorders, which collectively decrease the quality of life[12]. Therefore, a multidisciplinary approach is essential for the effective management of HS. Expert recommendations from the US and Canadian HS Foundations advise annual metabolic screening, including HbA1c or fasting glucose testing, in patients with HS who present with risk factors such as obesity, hypertension, dyslipidemia, etc.[24]. Clinicians should be encouraged to screen for comorbid conditions in patients beyond 40 years of age, those with high BMI, or those who present with sinus tracts or lesions. Ultimately, this case stresses the importance of recognizing that HS could be a direct sequela of underlying metabolic dysfunction.

From a surgical perspective, wide excision with flap reconstruction remains the gold standard for advanced HS, but the choice between single-stage versus staged procedures requires careful consideration of comorbidities, wound healing potential, and disease extent[25]. In this case, the decision for unilateral wide excision with flap reconstruction on right side, combined with incision and drainage, balanced the need for disease clearance with surgical safety in the context of poorly controlled diabetes and obesity. No wound healing complications were observed during early follow-up.

Prior studies suggest that HS is primarily driven by follicular occlusion and dysregulated immunity, with secondary bacterial colonization playing a contributory role[26]. Nevertheless, culture data may be useful for guiding perioperative antibiotic selection in recurrent or refractory cases.

This report presents the clinical course of a single patient, which limits the generalizability of findings. As the follow up period was relatively short, resulting in failure to assess long term outcomes and recurrence risk such as photographic documentation of prognosis and wound healing. Lack of culture limited the information of specificity of bacterial colonization. The patient perspective was brief and derived from routine clinical conversation, lacking an in-depth assessment of psychosocial impact. Moreover, detailed imaging records were limited due to typical nature of disease at presentation. Finally, as no control group was available for comparison, the efficacy of chosen surgical approach cannot be directly compared with other alternative treatments.

## Conclusion

This case emphasizes the need for stage-based management of HS and highlights the impact of metabolic comorbidities like diabetes. Patient outcomes can be improved by early diagnosis, timely surgical intervention, and metabolic control to minimize the chance of recurrence. Our case exemplifies how HS can serve as a sentinel presentation of undiagnosed diabetes, reinforcing the importance of metabolic screening in patients with severe disease. Future reports should emphasize the role of multidisciplinary care, involving dermatology, surgery, and endocrinology, to optimize outcomes.

## Data Availability

Data sharing is not applicable to this article as no datasets were generated or analyzed.
